# Loss of Endothelial Barrier Function in the Inflammatory Setting: Indication for a Cytokine-Mediated Post-Transcriptional Mechanism by Virtue of Upregulation of miRNAs miR-29a-3p, miR-29b-3p, and miR-155-5p

**DOI:** 10.3390/cells10112843

**Published:** 2021-10-22

**Authors:** Daniel Maucher, Birte Schmidt, Julia Schumann

**Affiliations:** University Clinic and Outpatient Clinic for Anesthesiology and Operative Intensive Care, University Medicine Halle (Saale), Franzosenweg 1a, 06112 Halle (Saale), Germany; Daniel.Maucher@uk-halle.de (D.M.); birteschmidt09@gmail.com (B.S.)

**Keywords:** endothelial barrier, miRNAs, tight junctions, adherens junctions

## Abstract

Dysfunction of the endothelial barrier plays a central role in the pathogenesis of both acute and chronic inflammatory processes such as sepsis or atherosclerosis. Due to attenuation of endothelial cell contacts, there is an increased transfer of blood proteins and fluid into the surrounding tissue, which relates to edema formation and distribution disorders. However, the mechanisms underlying these responses are not fully understood. In this study, we used human endothelial cells to mimic the loss of barrier function in an inflammatory milieu. We found that a weakened endothelial barrier after cytokine stimulation was accompanied by a significantly changed transcriptome. Apparent was a depletion of mRNAs encoding cell adhesion molecules. Furthermore, we found that cytokine treatment of endothelial cells induced upregulation of miR-29a-3p, miR-29b-3p, and miR-155-5p. miRNAs are known to negatively affect stability and translational efficiency of target mRNAs. Remarkably, miR-29a-3p, miR-29b-3p, and miR-155-5p have already been described to target the mRNAs of central tight and adherent junction proteins including F11 receptor, claudin 1, β-catenin, p120-catenin, and eplin. This taken together points to the existence of a posttranscriptional mechanism for expression inhibition of central adhesion proteins, which is triggered by inflammatory cytokines and mediated by miR-29a-3p, miR-29b-3p, and miR-155-5p.

## 1. Introduction

The endothelium, formed by endothelial cells, lines all blood vessels. In the intact vascular system, the endothelium represents a continuous barrier and actively regulates the distribution of substrate and fluid between blood and tissues. To achieve this balance, endothelial cells form tight junctions (TJs) and adherens junctions (AJs) with each other. TJs serve to seal the intercellular space and thus have a direct limiting effect on paracellular permeability [1,2]. AJs contribute to barrier stabilization by providing mechanical strength to the endothelial cell layer [1,2]. The contact of adjacent endothelial cells within TJs and AJs is ensured by transmembrane proteins, such as occludin (*OCLN*), F11 receptor (*F11R*), JAM-B (*JAM2*), JAM-C (*JAM3*), claudin 1 (*CLDN1*), claudin 5 (*CLDN5*), and VE-cadherin (*CDH5*) [1,2]. These proteins are associated with the cellular cytoskeleton via adhesion molecules such as α-catenin (*CTNNA1*), β-catenin (*CTNNB1*), γ-catenin (*JUP*), p120-catenin (*CTNND1*), vinculin (*VCL*), ZO-1 (*TJP1*), ZO-2 (*TJP2*), and eplin (*LIMA1*) [1,2]. The expression and distribution of TJ and AJ proteins are strictly regulated to ensure stability, integrity, and proper function of the endothelial barrier. It is worth noting that during inflammatory processes, these binding molecules are reorganized. Previous studies have shown that treatment of endothelial cells with cytokines alters both the amount and localization of TJ and AJ proteins, leading to intercellular gap formation and reduction of endothelial integrity [1,3,4]. The attenuation of endothelial cell–cell contacts is a physiological adaptation to facilitate the process of immune cell diapedesis into infected tissues. However, in systemic inflammatory processes such as sepsis or in the course of chronic inflammation, this inherently beneficial adaptive response shifts into a clinically significant maladaptation. Excessive, permanent and generalized activation of the endothelium occurs, which is accompanied by dysfunctionality [2,5,6,7,8]. Characteristic of this dysfunctionality is, among other things, a breakdown of barrier function.

Previous studies point at microRNA (miRNA)-mediated regulation of gene expression as a critical factor in deregulated endothelial function [9,10,11,12,13]. miRNAs are involved in the fine-tuning of gene expression. At the posttranscriptional level, miRNAs regulate the stability and translational efficiency of protein-encoding mRNAs [11,13,14,15]. The interaction between miRNAs and mRNAs is based on partial complementarity, so that a single miRNA can regulate the expression of hundreds of different mRNAs [11,14]. Indeed, disruption of miRNA-mediated regulation of TJ and AJ protein expression has already been associated with the formation of abnormal binding structures [16]. In an in vitro model of ischemic stroke, inhibition of miR-155 improved endothelial integrity and had a positive effect on claudin 1 and ZO-1 expression [12]. Additionally, miR-147b has been reported to impact endothelial barrier function owing to its ability to target the metalloproteinase ADAM15, which promotes increased endothelial permeability [17]. Taken together, these data suggest that miRNAs may play a critical role in endothelial function and vascular barrier integrity by affecting in a posttranscriptional manner the expression of adhesion molecules. Thus, the aim of the present study was to examine the possibility that miRNAs may act as (co)mediators in the loss of endothelial barrier function under inflammatory conditions.

## 2. Materials and Methods

### 2.1. Culturing and Stimulaton of Cells

The human cell line TIME (ATCC^®^ number CRL-4025) cultured at 37 °C and 5% CO_2_ in a humid atmosphere served as an in vitro model for microvascular endothelial cells. As recommended by ATCC^®^, basal microvascular endothelial growth medium (Provitro, Berlin, Germany) was supplemented with 5 ng/mL VEGF, 5 ng/mL EGF, 5 ng/mL FGF, 15 ng/mL IGF-1, 10 mM L-glutamine, 0.75 U/mL heparin sulfate, 1 µg/mL hydrocortisone hemisuccinate, 50 µg/mL ascorbic acid, 5% *v*/*v* FCS, and 12.5 µg/mL blasticidin. Cell stimulation was performed over a 24 h period by adding the cytokines IL-1β, TNF-α and IFN-γ at a concentration of 5 ng/mL each (all PeproTech, Hamburg, Germany).

### 2.2. Impedance Analysis

Barrier stability was monitored in a non-invasive, label-free manner by continuous impedance measurement using the microelectronic biosensor system for cell-based assays xCELLigence^®^ RTCA DP (ACEA Bioscience Incorporation via OLS, Bremen, Germany) according to manufacturer’s instructions. For xCELLigence measurements, TIME on gold electrode-coated plates (E-Plate 16 PET, OLS, Bremen, Germany) were placed in the xCELLigence RTCA DP system and real-time monitoring of transendothelial resistance was initiated for 48 h. Impedance was measured between gold electrodes in individual wells of the E-Plate and converted to the unit-free parameter cell index (CI) by the RCTA software (version 2.0; ACEA Biosciences Inc., San Diego, CA, USA). When a confluent monolayer was reached, the cytokine-free culture medium was replaced by a cytokine-containing one (5 ng/mL each of IL-1β, TNF-α, and IFN-γ). After 12 h of incubation, the medium was changed back to the cytokine-free culture medium.

Impedance analysis was performed with three biological replicates and two technical replicates tested in each group. Two-way analysis of variance (ANOVA) with correction for multiple comparisons (Sidak) was performed to determine significant differences in means, with a *p* value < 0.05 as an indicator of significant differences. GraphPad Prism 9 (GraphPad Software, La Jolla, CA, USA) was used as the statistical analysis software.

### 2.3. RNA Isolation

Total RNA was obtained using TRIzol-based standard isolation procedure following the manufacturer’s directions (Thermo Fisher Scientific, Dreieich, Germany). Quality determination of the extracted RNA was performed using NanoDrop spectrophotometer (Thermo Fisher Scientific, Dreieich, Germany) and Agilent Bioanalyzer (Agilent Technologies, Waldbronn, Germany). For subsequent procedures, only RNA with an absorbance quotient A260/280 >1.8 was used.

### 2.4. MRNA Expression (Transcriptome) Screening

mRNA-seq library preparation and deep-sequencing were performed by Novogene (UK) Company Limited (Cambridge, UK). Next generation sequencing (NGS) was carried out by means of an Illumina HiSeq2500 (San Diego, CA, USA). Three biological replicates were analyzed in every test group.

Differential gene expression analysis was conducted by the Core Facility Imaging, University Medicine Halle (Saale). In this analysis, low quality read ends as well as remaining parts of sequencing adapters were clipped off using Cutadapt software (https://cutadapt.readthedocs.org/ (accessed on 3 September 2021)). The processed sequencing reads were aligned to the human genome (UCSC GRCh38) using HiSat2 v2.1.0 (http://daehwankimlab.github.io/hisat2/ (accessed on 3 September 2021)) [18]. Removal of secondary alignments as well as filtering and indexing of alignments was performed using samtools [19]. FeatureCounts v1.53 (http://subread.sourceforge.net/ (accessed on 3 September 2021)) [20] was used for summarizing gene-mapped reads. Ensembl [21] was used as annotation basis. Differential gene expression was determined using the R package edgeR v3.26.8 (http://bioconductor.org/packages/release/bioc/html/edgeR.html (accessed on 3 September 2021)) [22] utilizing trimmed mean of M-values (TMM) normalization. Normalized count data subsequently were FPKM (fragments per million mapped reads) transformed. All generated RNA-seq data were deposited at Gene Expression Omnibus (GEO) repository.

### 2.5. MiRNA Expression Screening

NGS and NanoString technology were used in parallel to identify differentially expressed miRNAs. Three biological replicates were screened per test group.

Small RNA-seq library preparation, deep sequencing, and differential gene expression analysis were conducted by the Core Unit DNA, Leipzig University. NGS was carried out viaan Illumina HiScanSQ (Illumina Inc., San Diego, CA, USA). For differential gene expression analysis low-quality read ends and leftover sequencing adapters were truncated via Cutadapt software. Only sequences between 15 and 27 bases in length were analyzed. Reads were aligned to the human genome (UCSC GRCh38) and to mature sequences deposited in miRBase v21 by using the Bowtie aligner [23]. Removal of secondary alignments as well as filtering and indexing of alignments was performed using samtools [19]. The R/Bioconductor programming environment was used to determine the number of mapped reads using the ShortRead library with an acceptable error rate of 1 nt per mature miRNA sequence. Reads per million (RPM) and the TMM algorithm were used independently for data normalization. All generated small RNA-seq data were deposited at Gene Expression Omnibus (GEO) repository.

NanoString analysis was performed at the Institute of Human Genetics, Martin Luther University Halle-Wittenberg, Germany, with the nCounter FLEX Analysis System (NanoString Technologies, Hamburg, Germany). The nCounter human v3 miRNA expression assay was run according to the manufacturer’s instructions. The nSolverTM Analysis Software v3.0 (NanoString Technologies Inc., Seattle, WA, USA) was applied for data normalization. This included subtraction of background noise based on the negative controls of the miRNA assay, technical normalization based on the positive controls of the miRNA assay, and biological normalization based on the comparison of the samples with respect to the 100 most highly expressed miRNAs and the so-called positive ligation controls to compensate for potential differences in the amount of total RNA between the samples.

### 2.6. Droplet Digital PCR (ddPCR)

Complementary DNA (cDNA) was synthesized according to standard protocol of miRCURY LNA RT Kit (QIAGEN, Hilden, Germany), with spike-in RNA UniSp6 added to all samples as positive control. Subsequently, miRNA copy numbers were determined by applying droplet digital PCR technology (BioRad, Munich, Germany) according to the manufacturer’s standard protocols and using appropriate miRCURY LNA miRNA PCR assay primers (QIAGEN, Hilden, Germany) and ddPCR EvaGreen Supermix (Bio-Rad, Munich, Germany). Nucleic acid copy number per µl of a sample was measured using a QX200 ddPCR Droplet Reader (Bio-Rad, Munich, Germany) and converted to nucleic acid copy number per ng of RNA.

ddPCR reaction was performed with six biological replicates and two technical replicates tested in each group. An unpaired t-test was performed to determine significant differences in means, with a *p* value < 0.05 as an indicator of significant differences. GraphPad Prism 9 (GaphPad Software, La Jolla, CA, USA) was used as the statistical analysis software.

### 2.7. In Silico Analyses

To assess statistically significant, consistent differences in the transcriptome of cytokine-stimulated versus unstimulated endothelial cells, a gene set enrichment analysis (GSEA) was performed. GSEA was conducted by the Core Facility Imaging, University Medicine Halle (Saale) using the GSEA v3.0 software (UC San Diego and Broad Institute, San Diego, CA, USA) [24] and MSigDB v7.0 gene sets [25] thereby applying the pre-ranked test, 1000 permutations and the classical scoring scheme. Log2 fold changes of all protein coding genes’ expression determined by the differential expression analyses were used as input. A false discovery rate (FDR) < 0.05 was set as cut off criterion.

Transcription factor binding sites of differentially expressed miRNAs were identified utilizing the online database GeneCards v5.3 (https://www.genecards.org/ (accessed on 26 May 2021)) [26].

Target genes of differentially expressed miRNAs were identified utilizing the online databases miRWalk2.0 (http://zmf.umm.uni-heidelberg.de/apps/zmf/mirwalk2/index.html (accessed on 7 June 2021)) [27] and DIANA-TarBase v8 (https://carolina.imis.athena-innovation.gr/diana_tools/web/index.php?r=tarbasev8%2Findex (accessed on 7 June 2021)) [28].

Gene Ontology (GO) enrichment analysis was performed on the identified target genes of each miRNA using the online GeneOntology enrichment analysis and visualization tool (GOrilla) (http://cbl-gorilla.cs.technion.ac.il/ (accessed on 28 June 2021)) [29]. In order to identify GO processes that appear densely at the top or the bottom of the ranked list of target genes of a given miRNA, the GO enrichment analysis was carried out in the “single list mode”. A false *p* value < 0.01 was set as cut off criterion.

## 3. Results

### 3.1. Treatment with Proinflammatory Cytokines Attenuates Endothelial Expression of Molecules of Homophilic Cell–Cell Adhesion and Results in Loss of Endothelial Cell Layer Integrity

**Figure 1:**
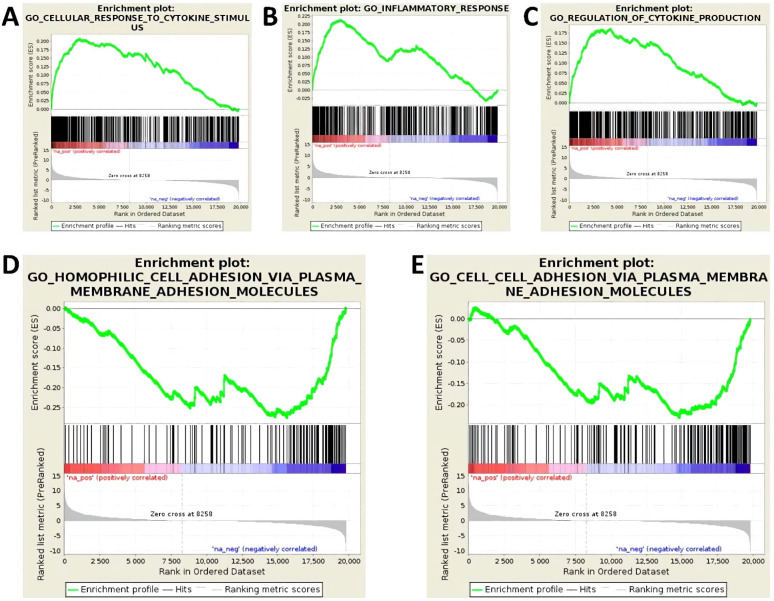
Human endothelial cells (cell line TIME, ATCC^®^ number CRL-4025) were evaluated following stimulation with 5 ng/mL each of IL-1β, TNF-α, and IFN-γ for 24 h and compared to untreated controls. Three biological replicates were tested in each group. Transcriptome data obtained by next generation sequencing (NGS) were gathered into a gene set enrichment analysis (GSEA). Shown are 5 enrichment plots, which correspond to defined gene sets. (**A**) GO-Cellular-Response-To-Cytokine-Stimulation, (**B**) GO-Inflammatory-Response, (**C**) GO-Regulation-Of-Cytokine-Production, (**D**) GO-Homophilic-Cell-Adhesion-Via-Plasma-Membrane-Adhesion-Molecules, (**E**) GO-Cell-Adhesion-Via-Plasma-Membrane-Adhesion-Molecules. Each plot shows the running Enrichment score (ES) for the gene set, which reflects the degree to which a gene set is over-represented at the top or bottom of a ranked list of genes. A positive ES indicates gene set enrichment at the top of the ranked list (=upregulation); a negative ES indicates gene set enrichment at the bottom of the ranked list (=downregulation).

Cytokine stimulation of endothelial cells is known to result in distinct functional adaptions. For visualization of transcriptome changes involved in this process, a GSEA was used. GSEA is a computational method to determine whether an a priori-defined set of genes displays significant and concordant differences in expression between two biological states [24,30]. In this study, mRNA expression of cytokine-stimulated versus unstimulated endothelial cells was compared. As shown in Figure 1, cytokine stimulation is accompanied with a marked inflammatory response. The expression of genes involved in the regulation of immune defense and cytokine production is clearly elevated (Figure 1A–C; FDR < 0.01 for all presented gene sets). In addition, GSEA provides evidence that the expression of membrane adhesion proteins mediating homophilic cell–cell adhesion is significantly reduced due to cytokine stimulation (Figure 1D–E; FDR 0.0 for both presented gene sets).

To gain a deeper insight, the integrity of the endothelial single cell layer was monitored utilizing the xCELLigence^®^ RTCA DP instrument. This approach allows for continuous, non-invasive, and label-free detection of cell responses in real-time through measurements of cellular impedance. The change in impedance is reported as a dimensionless parameter called cell index.

cell index = impedance at time point n − impedance without cells/nominal impedance value(1)

**Figure 2:**
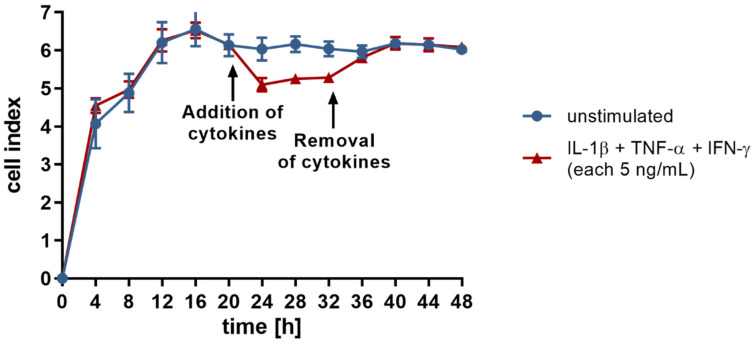
The cellular impedance of human endothelial cells (cell line TIME, ATCC^®^ number CRL-4025) was investigated in real-time using the xCELLigence^®^ RTCA DP instrument (ACEA Bioscience Incorporation). Changes in impedance are reported by the dimensionless parameter “cell index”. The blue and red data points symbolize cells cultured in basal cell culture medium versus medium containing 5 ng/mL each of IL-1β, TNF-α, and IFN-γ for a defined time, respectively. The arrows indicate the time points of the performed medium changes. Three biological replicates and two technical replicates were tested in each group. Data are shown as means ± standard deviation.

Under standard cell culture conditions, endothelial cells form close cell–cell contacts, as reflected by a high cell index of 6.1 on average (Figure 2). Cytokine treatment results in a sustained reduction of cell index (Figure 2; *p* < 0.001). Of note, this weakening of the endothelial barrier is completely reversed following removal of the stimulus through culture medium replacement. Both loss and restoration of the integrity of the endothelial cell layer occurs in less than 4 h after addition or removal of the cytokines (Figure 2). This observation points to the participation of a rapid regulatory mechanism, such as a miRNA-mediated posttranscriptional modulation of adhesion protein expression.

### 3.2. Activation of Endothelial Cells by Proinflammatory Cytokines Induces the Upregulation of miR-29a-3p, miR-29b-3p, and miR-155-5p

Multiple studies have investigated transcriptional control responses of endothelial cells under inflammatory conditions, but there is limited information focused on posttranscriptional regulatory events. For assessment of cytokine-mediated changes in endothelial miRNA expression, two screening approaches were performed in parallel to exclude false positive candidates at the earliest possible stage: the ligation-based Illumina next generation sequencing (NGS) and the hybridization-based NanoString technology. The Illumina method utilizes the sequencing-by-synthesis technology, which tracks the addition of labeled nucleotides as the nucleic acid strand is copied. The NanoString method is a variation of the DNA microarray approach, in which color-coded probes and microscopic imaging are used to detect and count formed transcripts. By doing so, seven miRNAs were identified (all upregulated), which (i) were detectable with an abundance >100 reads in both methods and (ii) showed a cytokine-induced altered expression rate >2-fold: miR-21-5p, miR-29a-3p, miR-29b-3p, miR-146a-5p, miR-155-5p, miR-181b-5p, and miR-221-5p.

**Figure 3:**
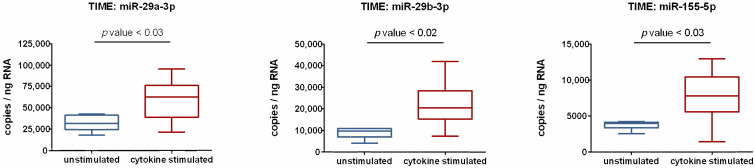
Human endothelial cells (cell line TIME, ATCC^®^ number CRL-4025) were evaluated following stimulation with 5 ng/mL each of IL-1β, TNF-α, and IFN-γ for 24 h, and compared to unstimulated controls. Absolute quantification of miRNAs from isolated total RNA was performed using ddPCR analysis. The QX 200 Droplet Digital PCR System (Bio-Rad) and specific LNA PCR primers (Qiagen) were used according to the manufacturer’s instructions. Based on the number of measured positive droplets, the system calculates the copy number of the target RNA in the total mixture, assuming a Poisson distribution. Since a defined amount of RNA sample is used in each ddPCR assay, the copy number per ng RNA can be determined. The box plots depict the median of determined miRNA expression levels, the lower and upper quantile, and the two extreme values. Each group included six biological replicates and two technical replicates. An unpaired t-test was used to identify significant differences. Statistical analysis was performed utilizing GraphPad Prism 9 (GaphPad Software, La Jolla, CA, USA). In all cases, *p* < 0.05 was assumed to indicate significant differences.

Next, screening results obtained with NGS and NanoString were subjected to PCR-based validation. Typically, RealTime PCR is used for this purpose; however, this approach only allows quantification relative to a housekeeping RNA, the expression of which is assumed to be unchanged. More detailed results can be obtained using droplet digital PCR (ddPCR). In this method, each sample is portioned into 20,000 droplets, thereby randomly distributing target and background DNA. Amplification of the target sequence takes place in each droplet by end-point PCR. Subsequently, positive droplets are counted to provide a precise quantification of the target sequence in the specimen. Thus, ddPCR enables the determination of relative expression changes as well as absolute quantification of miRNA copy number. Another advantage of this strategy is its independence from a reference RNA, which eliminates the risk of normalization-based data bias. Using ddPCR, three of the miRNAs studied were validated. As shown in Figure 3, ddPCR data clearly demonstrate that the expression of miR-29a-3p, miR-29b-3p, and miR-155-5p is subject to regulation by proinflammatory cytokines. Compared to unstimulated controls, cytokine-treated endothelial cells display significantly increased expression of these miRNAs. The copy number is elevated 1.9-fold for miR-29a-3p, 2.5-fold for miR-29b-3p, and 2.1-fold for miR-155-5p. It is also important to emphasize the high miRNA abundance of several thousand copies per ng of RNA (Figure 3), which strongly suggests that the observed increases have functional importance.

### 3.3. Expression of miR-29a-3p, miR-29b-3p, and miR-155-5p Is Subject to the Influence of Cytokine-Induced Signaling Cascades

miRNA expression is known to be affected by various physiological and pathological stimuli [31]. Endogenous factors, such as cytokines, induce signaling cascades that ultimately lead to the activation of specific transcription factors. These in turn mediate transcriptional regulation of miRNA expression via interaction with distinct transcription factor binding sites of miRNA coding regions of DNA. To visualize the signaling pathways and transcription factors involved in cytokine-mediated upregulation of miR-29a-3p, miR-29b-3p, and miR-155-5p, a search of the GeneCards v5.3 database was performed. The transcription factor binding sites listed in the database were matched with the transcription factors known to be induced by the cytokines IL-1β, TNF-α, and IFN-γ [32,33,34,35,36,37,38,39]. Table 1 provides an overview of the transcription factors that fulfill these conditional criteria. Not only is the absolute number of hits striking. It also indicates that different signaling pathways mediate the cytokine-induced change in endothelial miRNA expression jointly. Indeed, the simultaneous exposure of cells to IL-1β, TNF-α, and IFN-γ is known to induce primarily three signaling pathways: NFκB pathway, MAPK pathway, and JAK-STAT pathway [32,33,34,35,36,37,38,39]. Any of the three miRNAs expressed in an altered manner appears to be susceptible to mediators of each of these signaling pathways. In light of that in silico data, it seems reasonable to assume that in an inflammatory milieu there is a synergistic cytokine effect inducing upregulation of miR-29a-3p, miR-29b-3p, and miR-155-5p.

### 3.4. miR-29a-3p, miR-29b-3p, and miR-155-5p Upregulated by Cytokine Treatment Target Key Mediators of Endothelial Barrier Function

Interaction of miRNAs with (partially) complementary mRNAs typically decreases their stability and/or translational efficiency. Consequently, upregulation of miR-29a-3p, miR-29b-3p, and miR-155-5p is expected to be accompanied by posttranscriptional silencing of the specific target genes. To determine a possible causal link between the cytokine-induced alteration of the cellular miRNA profile and the observed reduction in expression of proteins of homophilic cell–cell adhesion, the corresponding target genes were identified by in silico analysis using the miRWalk2.0 and DIANA-TarBase v8 databases. Of note, numerous members of TJs and AJs are found in the target gene lists of these miRNAs (Table 2). Target genes of all three miRNAs that have already been experimentally validated include central transmembrane proteins, such as F11 receptor (*F11R*) and claudin 1 (*CLDN1*), as well as adhesion proteins, such as β-catenin (*CTNNB1*), p120-catenin (*CTNND1*), and eplin (*LIMA1*).

In a further step of analysis, the entire target gene lists gained from miRWalk2.0 and DIANA-TarBase v8 databases were subject to a GO enrichment analysis by GOrilla. Again, a clear association with cellular adhesion was found for all three miRNAs. This is reflected in the enrichment of the GO term “homophilic cell adhesion via plasma membrane adhesion molecules” (GO:0007156) for miR-29a-3p (FDR 0.0198) and miR-29b-3p (FDR 0.022) as well as of the GO term “regulation of cell–cell adhesion” (GO:0022407) for miR-155-5p (FDR 0.0125). The demonstration of cytokine-induced upregulation of miR-29a-3p, miR-29b-3p, and miR-155-5p, together with the fact, already documented in databases, that key mediators of endothelial cell contacts are targets of these miRNAs, provides a clear indication that the loss of endothelial barrier function in the inflammatory milieu may be due, at least in part, to a cytokine-mediated posttranscriptional mechanism.

## 4. Discussion

Exposure of endothelial cells to proinflammatory cytokines is associated with fulminant alterations in cell behavior. Previous studies of this working group show that in an inflammatory setting there is a significant increase in the expression of coagulation factors, such as vWF and TF, adhesion molecules, such as ICAM-1 and VCAM-1, cytokines, such as IL-6, IL-8, GM-CSF, and MCP-1 [40] as well as reactive oxygen species [41]. Another important adaptation is the local breaking of endothelial cell–cell contacts. This physiological response, which is also shown in the present study, enables the extravasation of immune cells from the blood into surrounding infected tissue. Systemic or chronic loss of endothelial barrier function, however, is of clinical relevance, for example, in sepsis or in the context of atherosclerosis, and contributes to the deterioration of the disease state [2,5,6,7,8]. This underscores the importance of approaches to prevent or treat such maladaptation. The present study focuses on a potentially useful but so far almost unexplored aspect: the posttranscriptional regulation of endothelial adhesion proteins.

Reduction of endothelial barrier integrity following exposure to the proinflammatory cytokines IL-1β, TNF-α, and IFN-γ was found to (i) be reversible and (ii) take place within just a few hours. This is suggestive of a posttranscriptional regulatory mechanism. And indeed, notable changes were found in the miRNA profile of endothelial cells after cytokine stimulation. Expression of miR-29a-3p, miR-29b-3p, and miR-155-5p proved to be significantly increased (about a doubling) in cytokine-activated endothelial cells. A notable feature of the present study is that absolute copy number of miRNAs was determined for the first time. The observed relative doubling of expression therefore translates into an absolute increase of approximately 27,500 copies of miR-29a-3p per ng of total RNA, approximately 13,150 copies of miR-29b-3p per ng of total RNA, and approximately 4000 copies of miR-155-5p per ng of total RNA. It is worth noting that these data are corroborated by previous studies. In particular, increased expression has previously been described for miR-155-5p as a consequence of cytokine stimulation of endothelial cells [42,43]. For miR-29b, an upregulation in response to the bacterial stimulus LPS has been reported [44]. Collectively, these observations can be attributed to the signaling cascades induced by inflammatory stimuli, such as cytokines. The performed mapping of cytokine-activated transcription factors with the transcription binding sites of miR-29a-3p, miR-29b-3p, and miR-155-5p yielded numerous matches. Taken together, the data from this and previous studies indicate that stimulation of endothelial cells by cytokines and subsequent activation of transcription factors leads to upregulation of miR-29a-3p, miR-29b-3p, and miR-155-5p, which is likely to be of physiological relevance affecting endothelial cell functionality.

The in silico analyses performed provide strong indications that miR-29a-3p, miR-29b-3p, and miR-155-5p are involved in the regulation of endothelial permeability. Not only does GO enrichment analysis point in this direction. Database records demonstrate that all three miRNAs directly target TJ and AJ proteins. The proposed mechanism of posttranscriptional inhibition of endothelial adhesion protein expression provides a plausible explanation for the decrease in mRNAs encoding homophilic cell–cell adhesion proteins detected by GSEA after cytokine treatment of endothelial cells. In light of this data, it seems reasonable to assume that miRNAs are involved in the regulation of endothelial barrier function.

## 5. Conclusions

Taken together, this study highlights the possibility that miRNAs, namely miR-29a-3p, miR29b-3p, and miR-155-5p, may be involved in the regulation of endothelial permeability. This observation offers a new insight into the mechanism of inflammation-induced endothelial barrier dysfunction. However, the description of a posttranscriptional mechanism also opens up a number of issues that need to be addressed in subsequent studies. To what extent do miRNAs affect the expression of individual TJ and AJ proteins? Is it possible to preserve these proteins from posttranscriptional expression inhibition and concomitantly to maintain endothelial barrier function via blockade of miR-29a-3p, miR-29b-3p, and miR-155-5p? Is it sufficient to inhibit one of these miRNAs or do all three need to be blocked? And most importantly, what about biological significance? This requires detailed studies in vivo. Not until then will we be able to assess whether miR-29a-3p, miR-29b-3p, and miR-155-5p may be suitable as drug targets to treat a dysfunctional endothelial barrier in systemic or chronic inflammation.

## Figures and Tables

**Table 1:** Transcriptions factors affecting expression of miR-29a-3p, miR-29b-3p, and miR-155-5p were identified using the database GeneCards v5.3.

Pathway	miR-29a-3p	miR-29b-3p	miR-155-5p
NFκB pathway	*RELA, RELB, NFKBIZ*	*NFKBIZ*	*RELB*
MAPK pathway	*JUN, JUNB, JUND, FOS, FOSL2, CEBPA, CEBPB, CEBPG, CREB1, ATF1, ATF2, ATF3, ATF4, BATF*	*JUND, FOS, FOSL2, CEBPA, CEBPB, CEBPG, ATF3, BATF*	*JUNB, JUND, CEBPA, CEBPB, ATF2, BATF*
JAK-STAT pathway	*STAT3, STAT5A, IRF2, IRF4, IRF9*	*STAT3, IRF9*	*STAT5A, IRF4*

ATF = cAMP depending transcription factor, BATF = Basic leucine zipper ATF-like transcription factor, CEBP = CCAAT enhancer binding protein, CREB1 = cAMP responsive element binding protein 1, IRF = Interferon regulatory factor, JUN/JUNB/JUND/FOS/FOSL2 = AP-1 subunits, NFKBIZ = NFκB inhibitor zeta, RELA = p65, STAT = Signal transducer and activator of transcription.

**Table 2:** Target genes of miR-29a-3p, miR-29b-3p, and miR-155-5p were identified using the databases miRWalk2.0 and DIANA-TarBase v8.

Target Genes	miR-29a-3p	miR-29b-3p	miR-155-5p
Validated by previous experiments	*F11R, CLDN1, CLDN5, CTNNB1, JUP, CTNND1, VCL, TJP1, LIMA1*	*F11R, CLDN1, CTNNB1, CTNND1, VCL, LIMA1*	*F11R, CLDN1, CTNNA1, CTNNB1, JUP, CTNND1, TJP1, TJP2, LIMA1*
Putative due to sequence analogies	*OCLN, JAM3, CDH5, CTNNA1, TJP2*	*OCLN, JAM3, CLDN5, CDH5, CTNNA1, JUP, TJP1, TJP2*	*OCLN, JAM2, JAM3, CLDN5, VCL*

CDH5 = VE-cadherin, CLDN1 = claudin 1, CLDN 5 = claudin 5, CTNNA1 = α-catenin, CTNNB1 = β-catenin, JUP = γ-catenin, CTNND1 = p120-catenin, F11R = F11 receptor, JAM2 = junctional adhesion molecule-B, JAM3 = junctional adhesion molecule-C, LIMA1 = eplin, OCLN = occluding, TJP1 = zona occludens 1, TJP2 = zona occludens 2, VCL = vinculin.

## Data Availability

Deep sequencing datasets generated and analyzed in the present study are available in the Gene Expression Omnibus (GEO) repository, accession numbers GSE132361 (miRNA screening) and GSE141957 (transcriptome screening).
